# Engineering Porous PET-RAFT Scaffolds with PLGA–Insulin Nanoparticles: Advancing Bone Tissue Regeneration Through Additive Manufacturing

**DOI:** 10.3390/polym18101184

**Published:** 2026-05-12

**Authors:** Fernando E. Rodríguez-Umanzor, Mauricio A. Sarabia-Vallejos, Nicolás F. Acuña-Ruiz, Scarleth A. Romero-De la Fuente, Nicolás A. Cohn-Inostroza, David Ortiz Puerta, Enrique Martínez-Campos, Juan Rodríguez-Hernández, Claudio A. Terraza Inostroza, Carmen M. González-Henríquez

**Affiliations:** 1 Departamento de Química, Facultad de Ciencias Naturales, Matemáticas y del Medio Ambiente, Universidad Tecnológica Metropolitana, Santiago 7800003, Chile; fernando.rodriguezu@utem.cl (F.E.R.-U.); nicolas.acunar@utem.cl (N.F.A.-R.); nicolas.cohn@utem.cl (N.A.C.-I.); 2 Programa PhD en Ciencia de Materiales e Ingeniería de Procesos, Universidad Tecnológica Metropolitana, Santiago 8940000, Chile; scarleth.romerod@utem.cl; 3 Facultad de Ingeniería, Universidad San Sebastián, Santiago 8420524, Chile; mauricio.sarabia@uss.cl; 4 Instituto Universitario de Investigación y Desarrollo Tecnológico (IDT), Santiago 8940000, Chile; 5 Biomedical Engineering School, Faculty of Engineering, Universidad de Valparaíso, Valparaíso 2362905, Chile; david.ortiz@uv.cl; 6 Millennium Institute for Intelligent Healthcare Engineering (iHEALTH), Santiago 7820436, Chile; 7 Group of Organic Synthesis and Bioevaluation, Instituto Pluridisciplinar, Universidad Complutense de Madrid, Associated Unit to the ICTP-IQM-CSIC, 28040 Madrid, Spain; emartinezcampos79@gmail.com; 8 Polymer Functionalization Group, Departamento de Química Macromolecular Aplicada, Instituto de Ciencia y Tecnología de Polímeros-Consejo Superior de Investigaciones Científicas (ICTP-CSIC), 28006 Madrid, Spain; jrodriguez@ictp.csic.es; 9 Research Laboratory for Organic Polymers (RLOP), Faculty of Chemistry and Pharmacy, Pontificia Universidad Católica de Chile, Santiago 7820436, Chile; cterraza@uc.cl

**Keywords:** 3D printing, PET-RAFT resin, bone tissue engineering, PLGA–Insulin nanoparticles, porous scaffolds

## Abstract

Multifunctional scaffolds that combine structural support with the controlled delivery of bioactive agents remain a major challenge in tissue engineering. To extend the use of these devices in biomedicine, 3D printing is presented as an alternative that enables the manufacture of complex devices tailored to each patient, thereby solving specific problems in a timely and efficient manner. In this study, porous 3D scaffolds were fabricated via digital light processing (DLP) using a PET-RAFT resin composed of 2-(dimethylamino)ethyl methacrylate (DMAEMA) and poly(ethylene glycol) diacrylate (PEGDA_575_). Sodium chloride (NaCl) was incorporated as a porogen, while insulin-loaded poly(lactic-co-glycolic acid) (PLGA) nanoparticles were embedded as osteoinductive agents. The printed constructs exhibited high-resolution, reproducible trabecular-like architectures, as confirmed by micro-computed tomography (micro-CT), with interconnected pores averaging 70.7 ± 24.7 μm and a total porosity of 57.0 ± 6.98%. Thermal and chemical analyses confirmed scaffold stability and controlled degradability. Cytocompatibility assays using MC3T3-E1, C2C12, hGMSCs, and C166-GFP cells showed viability above 80% after 7 days (ISO 10993-5). Insulin-loaded nanoparticles enabled sustained release, characterized by an initial burst followed by gradual release up to 72 h. Dynamic bioreactor culture enhanced cell adhesion and RUNX2 expression, confirming the osteoinductive potential of the hybrid scaffold for advanced BTE applications. This study introduces an innovative PET-RAFT-derived resin that combines structural reinforcement with spatiotemporal regulation of insulin release, offering a potential strategy for enhanced biomaterial tissue engineering and tailored therapeutic interventions.

## 1. Introduction

Bone defects that exceed the body’s intrinsic regenerative capacity remain a significant clinical challenge for the medical staff dedicated to the treatment and rehabilitation of skeletal tissue [1]. Traditional therapies, such as autografts and allografts, remain the clinical gold standard; however, they have significant limitations, including limited tissue availability, donor-site morbidity, immune rejection, and the risk of disease transmission [2]. These drawbacks have driven the search for alternative strategies to promote effective bone repair. Tissue engineering has emerged as a promising solution by combining scaffolds, cells, and bioactive factors to stimulate bone regeneration [3]. Within this framework, bone tissue engineering (BTE) offers a promising avenue for restoring the structure and function of damaged bone [4]. Among the wide range of biomaterials investigated, hydrogels have attracted particular attention due to their high water content, tunable mechanical properties, and ability to mimic the extracellular matrix (ECM) [5].

The development of scaffolds is currently focused not only on providing temporary three-dimensional support for cell adhesion and proliferation but also on designing systems capable of delivering biological and physical cues to promote osteogenesis. Over the past two decades, significant efforts have been devoted to designing scaffolds with interconnected porosity, tunable degradation rates, and mechanical properties that closely mimic those of native bone tissue [6]. For this purpose, conventional fabrication techniques currently employed, such as freeze-drying, salt leaching, or gas foaming, offer limited control over pore geometry and interconnectivity, often leading to heterogeneous structures [7]. In contrast, additive manufacturing (AM) technologies have revolutionized scaffold fabrication by enabling precise spatial control of architecture and multi-material printing. Moreover, AM allows the incorporation of bioactive agents during fabrication [8]. A critical feature of scaffolds for BTE is their porosity and interconnectivity, as these parameters regulate nutrient diffusion, vascularization, innervation, and new tissue ingrowth. For instance, Karageorgiou et al. demonstrated that scaffolds with well-controlled pore sizes significantly enhance osteogenesis [9], while Abbasi et al. further highlighted that highly porous architectures facilitate bone regeneration by promoting cell infiltration and vascular network formation [10].

While the architecture and mechanical properties of scaffolds are crucial, the material’s underlying chemistry dictates the biological response. The chemical composition and surface functionalities of a scaffold determine how proteins adsorb upon implantation, how cells adhere through integrin receptors, and how signaling pathways that regulate, among others, osteogenesis and angiogenesis are activated [11]. For example, Ji et al. demonstrated that incorporating nanoapatite into PLGA-based nanofibrous scaffolds improved biocompatibility and degradation while supporting osteogenic responses [12]. In this context, the synthesis of biocompatible photosensitive resins for additive manufacturing has attracted growing attention as a strategy to precisely tailor the physicochemical and biological features of scaffolds. Such resins can be engineered to minimize cytotoxicity, ensure predictable degradation, and provide functional groups for biomolecule attachment, thereby bridging material science and biological performance.

Recent advances in polymer chemistry have introduced controlled radical polymerization techniques, thereby expanding the design space of these resins. Notably, photoinduced electron transfer–reversible addition–fragmentation chain transfer (PET-RAFT) polymerization has emerged as a powerful approach to fabricate biocompatible resins with precisely controlled architectures [13]. PET-RAFT polymerization was selected over conventional free radical polymerization (FRP) due to its enhanced control over radical generation and network formation, which is critical for DLP-based fabrication. Unlike FRP, PET-RAFT enables the formation of a homogeneous crosslinked structure by minimizing premature termination, allowing spatiotemporal regulation for polymer growth, even under oxygen-tolerant conditions. This results in improved structural fidelity, reproducibility, and resolution of the printed scaffolds.

Additionally, PET-RAFT light-mediated activation facilitates seamless integration with 3D printing platforms, enabling in situ polymerization and microscale patterning. In parallel, the incorporation of a tertiary amine-containing methacrylate monomer is key to the system’s biological performance. Its protonation under physiological conditions generates a positively charged surface associated with antimicrobial, antiviral, and antifouling activities, due to electrostatic interactions with negatively charged microbial membranes. Importantly, these systems can maintain adequate cytocompatibility in biomedical applications, including studies using bone precursor cells, which are directly relevant for bone tissue engineering. Together, these features enable the design of multifunctional scaffolds that combine structural support with controlled biological functionality [14].

The PET-RAFT technology has emerged as a highly promising approach for developing photopolymerizable resins for advanced 3D printing, owing to its ability to produce “living” materials with exceptional resolution and structural precision [15]. This method enables the creation of scaffolds with hierarchical, linked porosity, thereby enhancing their applications in tissue engineering and in nutrient or bioactive substance transport. Recent evaluations and investigations have indicated that hydrogel-based scaffolds made from materials like alginate, GelMA, PEG, and chitosan exhibit superior biocompatibility, supportive cellular environments, and drug delivery potential; however, they often encounter challenges concerning mechanical stability, structural integrity, and long-term scaffold durability, frequently necessitating reinforcement with supplementary components or inorganic fillers.

Moreover, PET-RAFT enables the integration and regulated release of pharmaceuticals by modifying the network chemistry, internal morphology, and surface characteristics of the printed materials [16,17]. Collectively, these investigations underscore the promise of PET-RAFT for the fabrication of tailored, multifunctional biomedical devices with modifiable characteristics for pharmacological and tissue-regenerative applications.

Beyond its classical role in glucose regulation, insulin can stimulate osteoblast proliferation, collagen synthesis, and alkaline phosphatase activity, thereby supporting early bone matrix formation [18]. Studies have shown that insulin-loaded scaffolds enhance osteogenic differentiation of pre-osteoblastic cells and promote mineralization, suggesting their potential as osteoinductive factors [19]. Furthermore, embedding bioactive agents within hydrogel or polymeric matrices enables controlled and prolonged release, thereby preventing rapid degradation and ensuring localized bioactivity over extended periods [20]. By integrating insulin into chemically engineered scaffolds, it becomes possible to synergistically connect material chemistry and drug delivery to promote both structural and functional recovery. In contrast to traditional hydrogel systems, the current PET-RAFT-based DLP scaffold was particularly engineered to integrate targeted therapeutic administration with enhanced structural stability for bone tissue engineering applications.

In this context, this study aims to develop multifunctional scaffolds for bone tissue engineering (BTE) through DLP printing using a PET-RAFT-based photocurable resin. The selection of PET-RAFT polymerization enables controlled network formation and improved structural fidelity, while DLP printing allows the precise fabrication of complex 3D architectures. In addition, a porogenic agent (NaCl) was incorporated to generate an interconnected porous network, and insulin-loaded PLGA nanoparticles are introduced to provide localized bioactive functionality. The novelty of this work lies in the integration of controlled polymerization, hierarchical porosity, and drug delivery within a single 3D-printed scaffold system. Comprehensive characterization, including physicochemical, mechanical, and biological analyses, was performed to evaluate the performance of the scaffolds for bone regeneration applications. This approach provides a scalable and high-precision strategy for designing advanced functional biomaterials for BTE.

## 2. Experimental Section

### 2.1. Material and Equipment Description

#### 2.1.1. Material

The resins for DLP printing were formulated based on the monomer 2-(dimethylamino)ethyl methacrylate (DMAEMA, 98%) and the crosslinking agent poly(ethylene glycol) diacrylate (PEGDA, Mn: 575 g mol^−1^). The reaction mixture also included 2-(butylthiocarbonothioylthio)propionic acid (BTPA, >95%) as the RAFT agent, eosin Y (EY) as the photocatalyst, triethanolamine (TeOH) as the co-catalyst, and Orange G as a free radical absorber. All of these reactives were obtained from Sigma-Aldrich (St. Louis, MO, USA). After printing, the samples were washed with isopropanol. Sodium chloride (NaCl) was used as the porogen, which was later removed by leaching. Both compounds were obtained from Merck (Darmstadt, Germany).

The NaCl particles were pre-sorted using a set of mesh sieves with variable sizes: N° 50 (300 μm), N° 100 (150 μm), N° 200 (75 μm), and N° 300 (63 μm) (Gilson, Lewis Center, OH, USA, ASTM-U certified). A commercial biocompatible resin (Raydent Crown & Bridge, provided by Zortrax, Warsaw, Poland) was used as a control for the printability tests.

To assess in vitro biodegradability, the scaffolds were incubated in three different media: PBS supplemented with pig pancreas lipase Type II (100–200 U/mg) and antibiotics (penicillin and streptomycin), complete DMEM culture medium, and distilled water. All reagents were purchased from Sigma-Aldrich (St. Louis, MO, USA).

Direct cytocompatibility assays were carried out using pre-osteoblastic MC3T3-E1 cells (mouse strain C57BL/6, Sigma-Aldrich, St. Louis, MO, USA) and mouse pre-myoblast cell line C2C12 (ATCC CRL-1772, Manassas, VA, USA), as well as human gingival mesenchymal stem cells (hGMSCs), obtained as primary cells isolated from gingival tissue donated by the Faculty of Dentistry, Universidad de Chile, under approved ethical protocols. Indirect cytocompatibility of the extracts was assessed in mouse endothelial cells C166-GFP (ATCC CRL-2583, Manassas, VA, USA). All these cells were cultured in DMEM with 2-[4-(2-hydroxyethyl)-1-piperazinyl]ethanesulphonic acid (HEPES), penicillin, streptomycin (all from Sigma-Aldrich, St. Louis, MO, USA), and fetal bovine serum Gibco^TM^, from Thermo Fisher Scientific (Waltham, MA, USA). Cell viability and cell adhesion were studied using the AlamarBlue HS^®^ reagent, DAPI (4′,6-diamidino-2-phenylindole) staining, Rhodamine Phalloidin, and Hoechst 33342 (all from Thermo Fisher Scientific, Waltham, MA, USA). For immunofluorescence, the osteogenic transcription factor was detected using an Alexa Fluor^®^ 488–conjugated anti-RUNX2 monoclonal antibody (Cytoskeleton Inc., Denver, CO, USA). Samples were fixed with 4% paraformaldehyde (PFA), permeabilized with 0.5% Triton X-100, and mounted using an antifade medium containing 2.5% DABCO^®^ in PBS–glycerol (87% glycerol, 10% PVA, and 3% PBS)—all obtained from Sigma-Aldrich. For the synthesis of insulin-loaded PLGA nanoparticles, poly(D, L-lactide-co-glycolide) (PLGA 75:25, Mn: 66,000–107,000) was obtained from Sigma-Aldrich (St. Louis, MO, USA) and dissolved in acetone (Winkler Ltda, Santiago, Chile). The aqueous phase was prepared with a solution of the surfactant Pluronic^®^ F-68 and human insulin, both purchased from Sigma-Aldrich (St. Louis, MO, USA). Following synthesis, the nanoparticle suspension was handled and purified using a dialysis membrane (Spectra/Por^®^ 7, MWCO 10000, Repligen Corporation, Waltham, MA, USA).

#### 2.1.2. Equipment

The scaffolds were 3D-printed using a DLP printer model Inkspire from Zortrax (Warsaw, Poland). The printed pieces underwent post-curing and washing in a dual-station Mercury Plus 2-in-1 system (Elegoo Inc., Shenzhen, China).

The rheological properties of the synthesized resins were evaluated using a rotational rheometer (MCR 72, Anton Paar, Graz, Austria) with a parallel plate geometry (PP 25, 25 mm diameter). Viscosity measurements were performed using oscillatory shear tests, with the plate gap set to 250 μm and temperature controlled at 25 °C via a Peltier system (CoolPeltier™).

The wettability of the resins on the printing surface was assessed by contact angle measurements using a Theta Lite optical tensiometer (Attension-Biolin Scientific, Gothenburg, Sweden).

The chemical characterization of the resins was performed via Raman spectroscopy using a CRM-Alpha 300 RA system (WITec GmbH, Ulm, Germany) with a 532 nm Nd:YAG laser (50 mW), and by FTIR in ATR mode using a Nicolet iS5 spectrometer (Thermo Fisher Scientific, Waltham, MA, USA).

Surface and morphological characterization were performed using optical microscopy (Zeiss Axioscope 5) and a field-emission scanning electron microscope (FE-SEM), model GeminiSEM 360 (Carl Zeiss AG, Oberkochen, Germany), equipped with a Gemini 1 optics InLens detector, energy-dispersive X-ray spectroscopy (EDS), and an Ultim Max 40 detector (Oxford Instruments, High Wycombe, UK). Samples were coated with an 8 nm gold layer by sputter coating (model 108 AUTO, Cressington Scientific Instruments Ltd., Watford, UK) to improve surface conductivity.

The three-dimensional internal structure of the scaffolds was obtained using micro-computed tomography (micro-CT) with a SkyScan 1272 system (Bruker Co., Kontich, Belgium). Image reconstruction was performed with NRecon software (Bruker Co., Kontich, Belgium, version 2.0), and subsequent image processing and segmentation were performed with CTAn software (Bruker Co., Kontich, Belgium, version 1.16.4.1) to enable quantitative morphometric analysis.

Thermal analysis of the 3D materials was performed using thermogravimetric analysis (TGA) and differential scanning calorimetry (DSC) on a simultaneous thermal analysis system TGA/DSC1 from Mettler Toledo (Greifensee, Switzerland).

Mechanical properties were determined through uniaxial compression tests using a universal testing machine (ProLine Z005, Zwick-Roell, Ulm, Germany) equipped with a 2500 N load cell.

Biological studies of cytocompatibility on the 3D scaffolds were performed by absorbance measurements using a BioTek Synergy HTX microplate reader (Agilent Technologies Inc., Santa Clara, CA, USA). Furthermore, the cells were visualized using an inverted fluorescence microscope (model IX73, Olympus, Tokyo, Japan).

Insulin-loaded PLGA nanoparticles (PLGA/Insulin NPs) were characterized by dynamic light scattering (DLS) using a Zetasizer NanoS90 instrument (Malvern Instruments Ltd., Malvern, UK) and by UV-Vis spectrophotometry using a Specord 205 spectrophotometer (Analytik Jena, Thuringia, Germany). FE-SEM was also used to evaluate the morphology and distribution of the NPs within the scaffolds.

Finally, cell adhesion on porous 3D scaffolds functionalized with PLGA–Insulin nanoparticles was monitored using FE-SEM and confocal microscopy. Fluorescence imaging was performed using a Leica Stellaris 8 laser scanning confocal microscope (Leica Microsystems, Wetzlar, Germany). Z-stack images were visualized and processed using LAS X (Leica Application Suite LAS X software, version 4.12), and FIJI/ImageJ (version 1.54r) with the 3D Viewer and ClearVolume plugins.

### 2.2. Methods

#### 2.2.1. Synthesis of DLP Resin and Incorporation of Porogen Agent

For the formulation of a resin printable by DLP, a base mixture containing DMAEMA:PEGDA_575_ was selected and subsequently modified to be sensitive to PET-RAFT polymerization. To achieve this, DMAEMA and PEGDA_575_ were added as main monomers. At the same time, eosin Y (EY) was included as a photocatalyst, triethanolamine (TeOH) as a co-catalyst, Orange G as a free radical photoabsorber, and 2-(n-butylthiocarbonothioylthio)propionic acid (BTPA) as the RAFT agent [21]. The molar ratios and detailed purification procedures, as well as the preparation of NaCl particles used as the porogen, are described in the Supplementary Materials (Section S1). Following resin formulation, printability was evaluated by determining the Bond number and the contact angle between the resin and the metallic build plate.

#### 2.2.2. Design and 3D Printing of the Scaffolds

The scaffolds were designed using AutoCAD 2022 (Autodesk, Inc., San Francisco, CA, USA) and fabricated by DLP printing. Cylindrical architectures with vertically and horizontally aligned channels were produced using resin formulations with and without NaCl. Printing parameters and post-processing procedures, including washing, UV curing, and salt leaching, are detailed in the Supplementary Materials (Section S2).

#### 2.2.3. Characterization of Resin and 3D Scaffold

The chemical structures of the resin and the 3D scaffolds were monitored by Raman and ATR-FTIR spectroscopy after photocuring, leaching, and sterilization, with particular attention to the most relevant and characteristic bands of each sample. Raman spectra were acquired point-by-point with a spatial resolution of 100 nm using an Alpha 300 RA system (WItec GmbH, Ulm, Germany) equipped with a Nd:YAG laser (maximum power 50 mW at 532 nm). All Raman measurements were performed in triplicate to ensure reproducibility, and the reported values are expressed as mean ± standard deviation. The polymerization degree was determined by calculating the area under the peak corresponding to the carbon–carbon double bond (C=C, 1635–1640 cm^−1^) (Equation (1)), normalized with respect to the reference band of the carbonyl (C=O) group located between 1715 and 1725 cm^−1^, which remains constant during the photopolymerization process [22]. In addition, ATR-FTIR spectra were obtained between 4000 cm^−1^ and 500 cm^−1^, with a resolution of 4 cm^−1^.

(1) $$C\nu =1-\frac{{\left(I_{C=C}\right)}_{p}}{{\left(I_{C=C}\right)}_{l}}$$

where (I_C=C_)_p_ is the area under the vibrational band of the C=C group after polymerization, and (I_C=C_)_l_ is the corresponding area for the liquid resin. To avoid interference from baseline tails or overlapping peaks, the selected spectral region (1800–1600 cm^−1^) was carefully analyzed, confirming that the bands were well resolved and exhibited a high signal-to-noise ratio (S/N ≥ 100). Local baseline correction and peak area integration were performed in Origin (OriginLab, version 8.5.1). The reported values were limited to three significant figures, consistent with the spectral signal-to-noise ratio.

**Figure 1:**
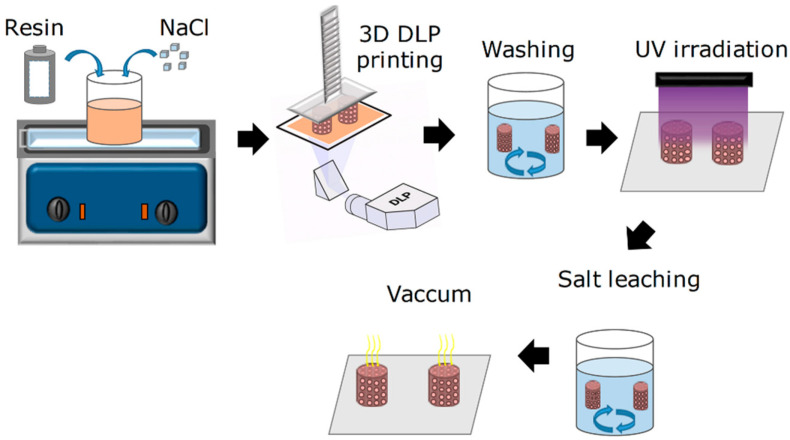
Schematic representation of the methodology used for the fabrication of porous 3D scaffolds.

After confirming a high degree of polymerization and the effective consumption of C=C bonds following printing, post-curing, and sterilization, the characterization was extended to assess how scaffold architecture and porosity influence the physicochemical and functional properties of the 3D-printed constructs. As schematically illustrated in Figure 1, porous scaffolds were obtained by incorporating a sacrificial porogen into the photocurable resin, followed by 3D printing, porogen removal, and subsequent structural, thermal, and mechanical evaluation. Sodium chloride (NaCl) was employed to generate interconnected microporosity within the polymeric network. Detailed experimental procedures related to porogen incorporation, leaching, and post-processing analyses are provided in the Supplementary Materials (Section S3).

#### 2.2.4. Enzymatic Degradation Study of Porous 3D Scaffolds

For the in vitro enzymatic degradation tests, porcine pancreatic lipase (Type II) was used as a model ester hydrolase. Although its physiological substrate is triacylglycerol, this enzyme is widely employed to catalyze the hydrolysis of ester linkages in aliphatic polyesters (e.g., PLA, PCL) and is therefore commonly used in biomaterial degradation studies [23]. Samples designated for enzymatic degradation were cut into equal parts and sterilized in ethanol for 30 min to ensure aseptic conditions. Subsequently, they were dried in a vacuum chamber for 24 h (10^−2^ torr). The degradation assay was performed following the protocols described by Hsieh et al. [24] and Lin et al. [25]. For this purpose, an enzymatic solution was prepared by dissolving porcine pancreatic lipase type II (20 U/mL) in PBS and filtering it through a sterile filter before use. Triplicate samples were placed individually in a 12-well cell culture plate, with 4 mL of the enzymatic solution in each well. Incubation was conducted at 37 °C, with the solution renewed every two days. Samples were retrieved for analysis at the following time points: 1, 1.5, 3, 5, 7, 10, 15, 21, and 30 days. After retrieval, the samples were washed with distilled water and dried again to calculate mass loss (Equation (2)).

(2) $$Weight\;loss\;\left(\%\right)=\left(\frac{m_{0}{-m}_{t}}{m_{0}}\right)\times 100\;$$

where m_0_ corresponds to the initial mass of the scaffold before treatment, and m_t_ is the mass of the scaffold after each immersion period. In addition to gravimetric analysis, selected samples from 1, 3, 5, 7, 15, and 30 days were examined by FE-SEM and analyzed by ATR-FTIR to evaluate morphological and chemical surface changes induced by enzymatic action. The ATR-FTIR spectra obtained during the enzymatic degradation assays were linearized and normalized using the band at 2868 cm^−1^, corresponding to the symmetric stretching vibrations of the methylene (–CH_2_) group. This signal was selected because it remains unaltered throughout the enzymatic degradation process, as it does not involve hydrolysis-susceptible bonds, thereby allowing correction of instrumental variations and quantitative comparison of the spectra collected at different degradation times [26].

#### 2.2.5. Cytocompatibility Assays of 3D Scaffolds and Extracts

The scaffolds were incubated in 300 μL of HEPES-supplemented DMEM (10 mM) at 37 °C, 1 atm, and 5% CO_2_ for 7 days, with medium renewal every 3–4 days. Due to the limited well size (<8 mm, 3.4 mL), each scaffold was sectioned into eight equal parts. Before cell seeding, the fragments were rinsed and immersed in 200 μL of PBS for 24 h. MC3T3-E1, C2C12, and hGMSCs cells were maintained in DMEM enriched with 10% FBS, 10 mM HEPES, 100 U/mL penicillin, 100 µg/mL streptomycin, and 2.5 µg/mL Amphotericin B. For the assay, 5 × 10^4^ cells were seeded onto polymeric disks measuring 10 mm in diameter and 3 mm in height, each placed in individual wells of a 24-well culture plate. The culture medium was replaced every 3 days. Cell viability was evaluated at days 1, 3, and 7 using the AlamarBlue HS^®^ assay. After 2 h of incubation with the reagent at 37 °C in a humidified incubator with 5% CO_2_, the supernatant was collected, and fluorescence was measured using a microplate reader at excitation/emission wavelengths of 560/590 nm. Leached scaffolds were also evaluated using an in vitro cytocompatibility test in accordance with ISO 10993-5 [27], the standard for the biological evaluation of medical devices [28]. In this case, a sterilized sample was placed in a 24-well plate with 3 mL of complete DMEM culture medium, alongside a control consisting of an empty well with the same volume of medium. The plate was incubated at 37 °C with 5% CO_2_ for 24 h to simulate physiological conditions. After incubation, three 500 µL aliquots were extracted and transferred to a 12-well plate (1:1 dilution), each well containing 500 µL of culture medium with GFP-labeled C166 endothelial cells, previously seeded at an initial density of 43,000 cells/cm^2^ and cultured for 24 h.

#### 2.2.6. Synthesis and Incorporation of PLGA–Insulin Nanoparticles into 3D Scaffolds

Following the fabrication and characterization of the porous 3D scaffolds, insulin-loaded PLGA nanoparticles (NPs) were incorporated into their cavities as osteoinductive agents. NPs were synthesized via the solvent-emulsion diffusion method described by Santander et al. [29], using PLGA, acetone, Pluronic^®^ F-68, and human insulin. The mixture was sonicated in 3 stages, followed by final dilution in deionized water. NPs’ size was measured by dynamic light scattering (DLS), and morphology was examined by FE-SEM. UV-Vis spectroscopy was used to confirm insulin loading. The scaffolds were immersed in the NP suspension under gentle stirring for 2 h at room temperature, then dried for 24 h, and analyzed again by FE-SEM to confirm NPs inclusion in the scaffolds. To verify that NPs incorporation did not compromise mechanical performance, uniaxial compression tests were repeated as described in Supplementary Materials (Section S3). Insulin release was assessed using UV-Vis spectroscopy over time in 10 mM PBS (pH 7.4). Scaffolds were placed in 10 kDa dialysis membranes and incubated in 10 mL PBS at 37 °C with mild agitation (30 RPM). Aliquots were collected at 6, 12, 24, 36, and 48 h, and insulin concentrations were quantified using a calibration curve based on the Beer–Lambert law [30].

#### 2.2.7. Cell Adhesion and Proliferation on 3D Scaffold with Nanoparticles in a Bioreactor

The hGMSCs cells from primary culture (passage 7, P7) were used to assess cell adhesion onto 3D-printed biomaterial scaffolds. The dynamic culture system consisted of sterile 100 mL Erlenmeyer flasks containing DMEM supplemented with 10% heat-inactivated FBS, 10 mM HEPES, and 1× antibiotic–antimycotic, providing final concentrations of 100 U/mL penicillin, 100 µg/mL streptomycin, and 0.25 µg/mL amphotericin B. Each flask was sealed with a sterile 0.2 µm filter cap and placed on a flat magnetic stirrer at low stirring speed. Biomaterial scaffolds were suspended from the cap using sterile 3-0 silk sutures. A total of 5 × 10^6^ hGMSCs were statically seeded onto each scaffold and incubated for 3 h to allow initial attachment. After this period, the flasks were placed in dynamic culture conditions at 25 RPM for 24 h at 37 °C in a humidified 5% CO_2_ incubator [31]. After incubation, samples were gently rinsed with PBS and fixed with 4% PFA for 15 min at room temperature. Fixed samples were washed twice with PBS and permeabilized with 0.5% Triton X-100 in PBS for 5 min. F-actin was stained using rhodamine phalloidin (Amanita phalloides, Cat. #PHDR1, Cytoskeleton, Inc., Denver, CO, USA). A 100 nM working solution was prepared from the 14 µM methanolic stock in PBS, and samples were incubated for 30 min at room temperature, protected from light. After three PBS washes, nuclei were counterstained with Hoechst 33342 at approximately 5 µg/mL in PBS. Samples were incubated for 15 min at room temperature, washed twice with PBS, and prepared for imaging. After staining, the scaffolds were transferred to 24-well plates and mounted using an antifade medium composed of PVA supplemented with 2.5% 1,4-diazabicyclo [2.2.2] octane (DABCO^®^) in PBS–glycerol (87% glycerol, 10% PVA, 3% PBS, *w*/*v*). To reduce light scattering and improve imaging depth, scaffolds were incubated in 80% glycerol (*v*/*v* in PBS) for 2 h at room temperature before imaging.

Statistical analysis was performed using OriginPro 2021. Data are presented as mean ± standard deviation. Statistical significance was evaluated using one-way ANOVA followed by Tukey’s HSD post hoc test; *p* < 0.05 was considered statistically significant. Additional details, including normality and variance tests, are provided in The Supplementary Materials (Section S4).

## 3. Results and Discussions

The concentrations of the resin components were selected based on recent findings from our research group. Sarabia-Vallejos et al. [22] reported the synthesis of a resin formulation designed to work via PET-RAFT polymerization using a DLP printer with a 525 nm light source. Herein, different RAFT agents, a photoinitiator, and a photoabsorber are employed to optimize the formation of the final 3D-printed part.

### 3.1. Design, Chemical–Physicochemical Characterization, and Printability of the Resin

Resin characterization began by evaluating its suitability for DLP printing processes. Key parameters, such as viscosity and surface tension, are critical for determining printability [32,33]. In this context, it has been reported that the zero-shear dynamic viscosity (µ_0_) should not exceed 5 × 10^3^ mPa·s, as higher values hinder printing by impeding the polymerization of new layers on top of the previous one [34]. Additionally, the Bond number (B_o_) is a dimensionless parameter used to compare the influence of gravitational forces with that of surface tension. In this case, low B_o_ values (below 1) are desired because they indicate a stable liquid bridge between the build plate and the resin surface, thereby improving printability. The dynamic viscosity, surface tension, density, and B_o_ values obtained for both commercial and synthesized resins are summarized in Table S1. The dynamic viscosity (µ_0_) was measured at low shear rates to represent conditions during the printing process (DLP printing occurs at low shear rates). The Bond number (B_0_), calculated from the measured density and surface tension, was found to be less than 1 in both cases [32], indicating the formation of stable liquid bridges [35]. Furthermore, the viscosity values remain within the acceptable range, indicating the resin’s printability under DLP conditions. This is corroborated by the successful fabrication of scaffolds exhibiting good print fidelity, interlayer adhesion, and preservation of the designed architecture. In general, rheological parameters are temperature-dependent and follow a linear Arrhenius relationship, so it is important to actively control temperature during printing. In our case, the temperature was maintained at 30 °C during printing using a heating system located in the printing chamber.

Additionally, another key factor that significantly affects DLP printing is the wettability between the resin and the build platform. An indirect way to evaluate this interaction is through contact angle measurements between the liquid resin and the metallic substrate. Lower contact angles indicate improved resin spreading on the metallic surface, favoring interfacial adhesion and homogeneous layer formation during printing. The contact angles measured for both commercial and synthesized resins were below 20°, indicating favorable wettability on the metallic build plate (Figure S1, Supplementary Materials), consistent with the successful fabrication of scaffolds under DLP conditions.

**Figure 2:**
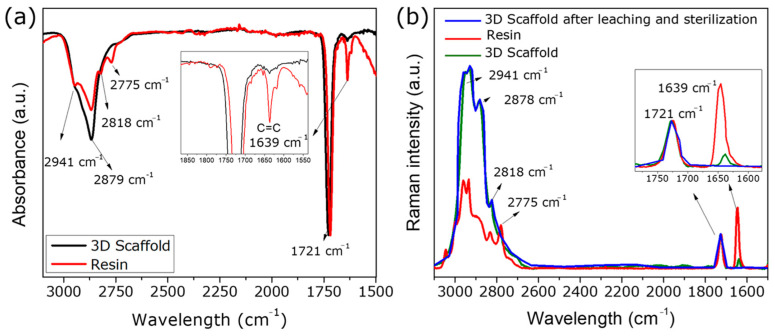
(**a**) ATR-FTIR spectra, and (**b**) Raman spectra of the liquid (unpolymerized resin) and solid 3D-printed scaffold. The Raman dataset additionally includes the 3D scaffold after the leaching and sterilization process.

Following physicochemical characterization and validation of the resins for DLP printing, chemical analyses were performed before and after 3D printing. ATR-FTIR and Raman spectroscopy were used to analyze the resin in two states: liquid resin (unpolymerized) and a solid printed part (3D-printed and post-cured). These analyses aimed to assess the extent of polymerization by monitoring the consumption of C=C bonds. In the ATR-FTIR spectrum shown in Figure 2a, characteristic signals of the resin components, i.e., PEGDA_575_ and DMAEMA, could be detected. These include a band between 2941 cm^−1^ and 2879 cm^−1^, corresponding to the asymmetric and symmetric stretching vibrations of –CH_3_ and –CH_2_ groups, respectively, and a peak at 1721 cm^−1^ attributed to the stretching of the carbonyl group (C=O) [36]. Since the carbonyl signal remains constant and undergoes little change during polymerization, it was used to normalize the spectra in both Raman and ATR-FTIR analyses. Additionally, distinctive peaks corresponding to the DMAEMA moiety were identified. Specifically, the bands at 2818 cm^−1^ and 2775 cm^−1^ were assigned to the C–H stretching vibrations of the R–N(CH_3_)_2_ group present in DMAEMA [37].

The Raman spectral characterization revealed consistent signals in both the liquid resin (unpolymerized) and the solid 3D-printed scaffold (3D-printed and post-cured; Figure 2b), corroborating the observations from ATR-FTIR. As seen in both ATR-FTIR and Raman spectra, a significant decrease in the intensity of the band at 1639 cm^−1^—associated with the aliphatic stretching vibrations of the C=C bond from methacrylate and acrylate groups—was detected. This band is directly correlated with the polymerization degree, as described by Equation (1). Based on these findings, a conversion of 87 ± 3.4% was obtained, indicating that approximately 13% of the monomer remained unreacted after printing and post-curing. Subsequently, the same 3D-printed part was subjected to leaching and sterilization for cytocompatibility assessment and then characterized by Raman spectroscopy (blue line). These measurements aimed to evaluate the presence of residual unsaturated species (C=C). No detectable signals were observed around 1638 cm^−1^ within the sensitivity of the technique, indicating that any remaining unreacted monomeric species are below the detection limit after post-processing. This suggests that the leaching and sterilization steps effectively reduced residual monomers to levels unlikely to interfere with the biological response. Furthermore, no significant changes were observed in the overall spectrum, indicating that the material remained structurally stable after both processes.

### 3.2. Printing and Characterization of Porous Scaffolds

**Figure 3:**
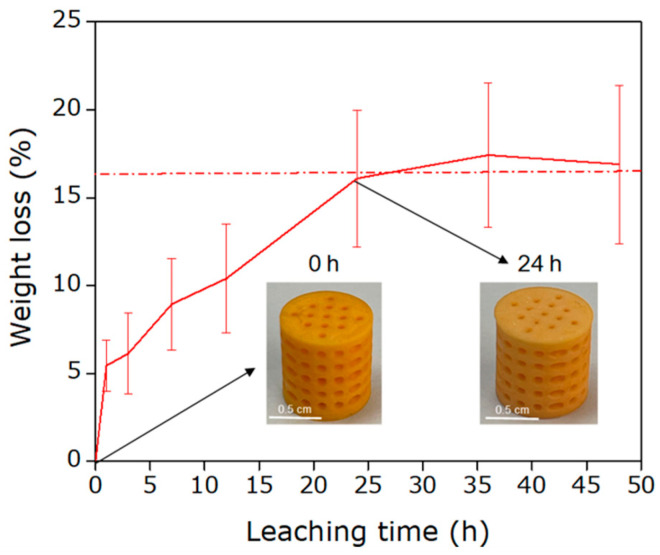
Evolution of weight loss as a function of the leaching time of the porogenic particles in the 3D-printed structures. Two protographies of the printed parts at 0 and 24 h is showed.

After evaluating the printability and chemical properties of the resin, the proposed formulation was tested using a DLP printer. A light exposure time of 120 s per layer and a layer height of 0.025 mm (Z-resolution) were employed. This exposure time was experimentally optimized by evaluating print fidelity, interlayer adhesion, and mechanical integrity of the printed parts, observing that shorter exposure times led to incomplete curing, structural defects, and poor mechanical stability. Under these conditions solid cylindrical models with a diameter of 1 cm and a height of 1 cm were fabricated, along with cylindrical models containing channels (Figure 3). Once the printing parameters for the proposed resin were standardized, NaCl particles with a crystal size of 63–75 µm were incorporated at a concentration of 15 wt.%, following to proportions previously reported by our research group [38]. Higher concentrations (>15 wt.%) led to faster particle sedimentation within the resin, resulting in a non-homogeneous distribution of the porogen agent and less uniform printed structures. Printing was carried out using the same parameters as before, with the process paused and mixed every 20 min to prevent particle sedimentation.

The leaching process was initially evaluated in Milli-Q water under constant agitation. The samples were weighed in triplicate before leaching and after 1, 3, 7, 12, 24, 36, and 48 h. As shown in Figure 3, the weight loss stabilized after 24 h, reaching approximately 16.5 ± 1.15 wt.%. Considering that the nominal NaCl content incorporated as a porogen was 15 wt.%, the slightly higher mass loss was attributed to the simultaneous removal of soluble species, such as unreacted monomers and low-molecular-weight oligomers, during leaching, indicating that the leaching process not only removes the salt but also “cleans” the 3D-printed structure by removing unwanted traces from the synthesis and printing processes.

**Figure 4:**
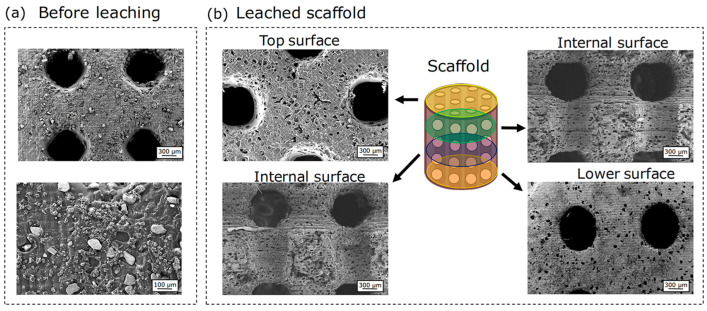
FE-SEM images of DMAEMA: PEGDA_575_-based scaffolds: (**a**) before leaching and (**b**) after leaching for 24 h.

Figure 4 shows FE-SEM micrographs of the scaffolds before leaching (Figure 4a) and after 24 h of leaching (Figure 4b). The final sample was sectioned into three equal parts to visualize both the external surfaces and the internal structure of the material. As shown in Figure 4a, the particles are clearly distinguishable in the FE-SEM images and exhibit a homogeneous distribution throughout the sample in different directions. Additionally, it is essential to note that the internal channels remained largely intact, with no evident signs of material detachment or deformation induced by the lixiviation procedure.

Conversely, pores were observed throughout all internal and external layers of the material (Figure 4b), which is directly associated with the channels facilitating the penetration of the aqueous solution into the scaffold cavities. By analyzing these micrographs, the pore size distribution of the 3D-printed structure was determined by measuring approximately n ≈ 150 pores from multiple regions of the scaffold using image analysis software (FIJI/ImageJ, version 1.54r). The average pore size was 63.6 ± 20.5 µm (Figure S2, Supplementary Materials). The relatively broad distribution reflects the inherent variability of the porogen-based fabrication process. This result is consistent with the mesh size of the sieves used during the preparation of the NaCl microparticles (mesh N° 200 and N° 300, corresponding to 63–75 µm).

**Figure 5:**
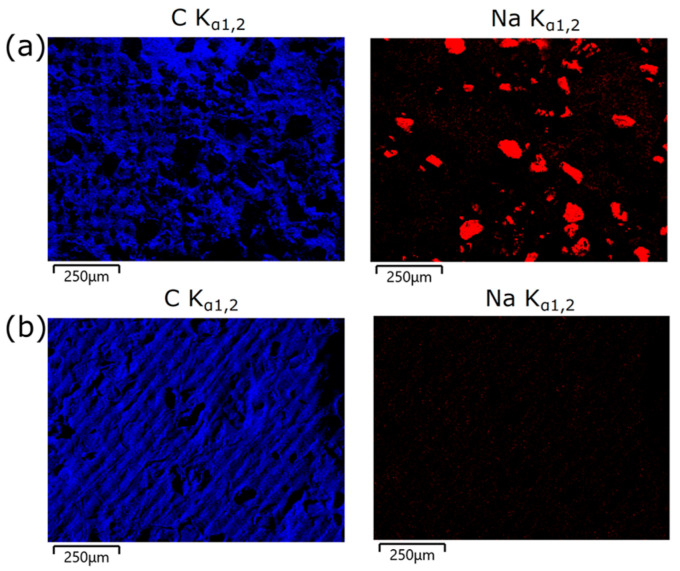
EDX elemental analysis of the DMAEMA:PEGDA_575_-based samples containing 15% NaCl: (**a**) before leaching, and (**b**) after leaching.

To confirm whether the salt had been fully leached after 24 h, energy-dispersive X-ray spectroscopy (EDX) analysis was performed on different regions of the sample. The EDX results (Figure 5a) showed two distinct signals: those corresponding to sodium atoms, indicative of the presence of NaCl particles, and signals related to carbon atoms, representative of the organic matrix background. As shown in Figure 5b, the leached samples showed a sodium content below 0.2%, indicating that the leaching process was effective.

**Figure 6:**
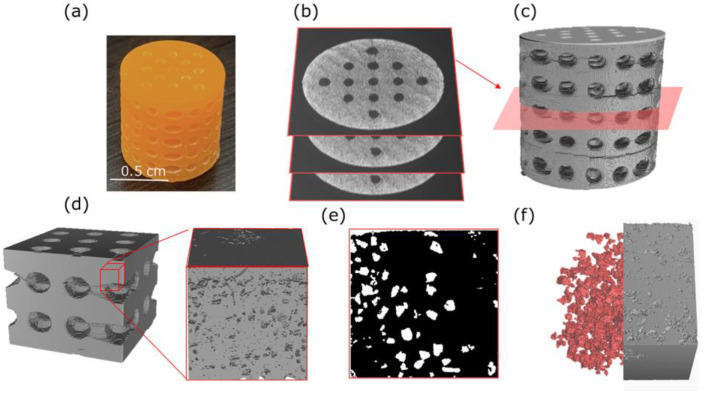
Schematic representation of the methodology used for the structural analysis of porous 3D scaffolds by micro-CT. (**a**) Photograph of the 3D-printed scaffold, (**b**) Set of multiple tomographic slices acquired from the micro-CT data, (**c**) 3D reconstruction from the slices, (**d**) Representative Volume Elements (RVE) selected from different regions of the sample, (**e**) Segmented image of a representative RVE slice (polymer matrix in black and pores in white), and (**f**) 3D representation of the segmented pores (red) and the resin matrix (gray) within each RVE.

To further elucidate the structural effects induced by the leaching process on the 3D scaffolds (Figure 6a), micro-computed tomography (micro-CT) analysis was performed. This technique enabled the acquisition of tomographic slices of the sample from multiple angles (Figure 6b), thereby facilitating the reconstruction of its internal 3D architecture (Figure 6c). Using these reconstructed images, several representative volume elements (RVEs) were selected from different sectors of the cylinder to determine local porosity, pore volume, and the Euler number [39]. Subsequently, a 3D morphological “opening” filter (round kernel, radius = 1) was applied to mitigate typical imaging artifacts, including noise and interface identification caused by object proximity or X-ray interactions.

On the other hand, the presence of non-leached salt residues can cause X-ray diffraction during microtomography, generating noise and false objects in the 3D image, thereby compromising segmentation accuracy and structural analysis. The processed image series (Figure 6d) was employed to quantify morphological features, including pore size and local porosity. As shown in Figure 6d, the generated pores were homogeneously distributed throughout the structure. To quantify these quantitative analyses, 10 RVE cubes were selected at evenly spaced intervals along the vertical axis of the printed cylinder. These RVEs were used to calculate local porosity using Equation (S2) (Supplementary Materials). The obtained values yielded an average porosity of 14.6 ± 2.3% *v*/*v*, which exceeds the initially incorporated salt content (8.5% *v*/*v*). This discrepancy can be attributed to several structural and process-related factors, including the coalescence of adjacent pores during the leaching process and the partial sedimentation of salt particles during printing [38,40]. In addition, the extraction of residual soluble species, such as unreacted monomers or low-molecular-weight oligomers, may contribute to an increase in the overall void fraction.

Additionally, the CAD-designed channels contributed to the structure’s total porosity of 57.0 ± 6.98%. This value lies within the lower end of the typical porosity range for trabecular bone (50–90%) and, although lower than that of high-porosity scaffold designs, it is sufficient to support essential biological processes such as nutrient diffusion, cell infiltration, and angiogenesis, provided that the pore connectivity is maintained. In this regard, morphological analysis revealed a highly interconnected 3D network, as evidenced by a negative Euler number (Eu.N = −3471), characteristic of systems with multiple internal communication pathways between cavities [41]. The inclusion of CAD-designed channels partially compensates for the lower porosity by providing predictable, fluid-conducting pathways that may enhance internal perfusion and promote uniform cellular colonization of the scaffold.

Finally, pore size was estimated by three-dimensional analysis of the segmented images, with spheroids fitted to the cavities using the expectation–maximization (EM) algorithm from Lesouple et al. [42]. The resulting diameters were processed, yielding an average pore size of 70.7 ± 24.7 μm (Figure S3), consistent with the FE-SEM observations (Figure 4) and with the initial particle size of the NaCl used as the porogen. In addition, a segmentation algorithm was applied to detect residual salt particles, revealing a remaining fraction of 0.5% *v/v* of NaCl grains. This value is small enough to be considered negligible and aligns with the EDX analysis (Figure 5).

### 3.3. Thermal Behavior of the 3D = Printed Scaffolds

**Figure 7:**
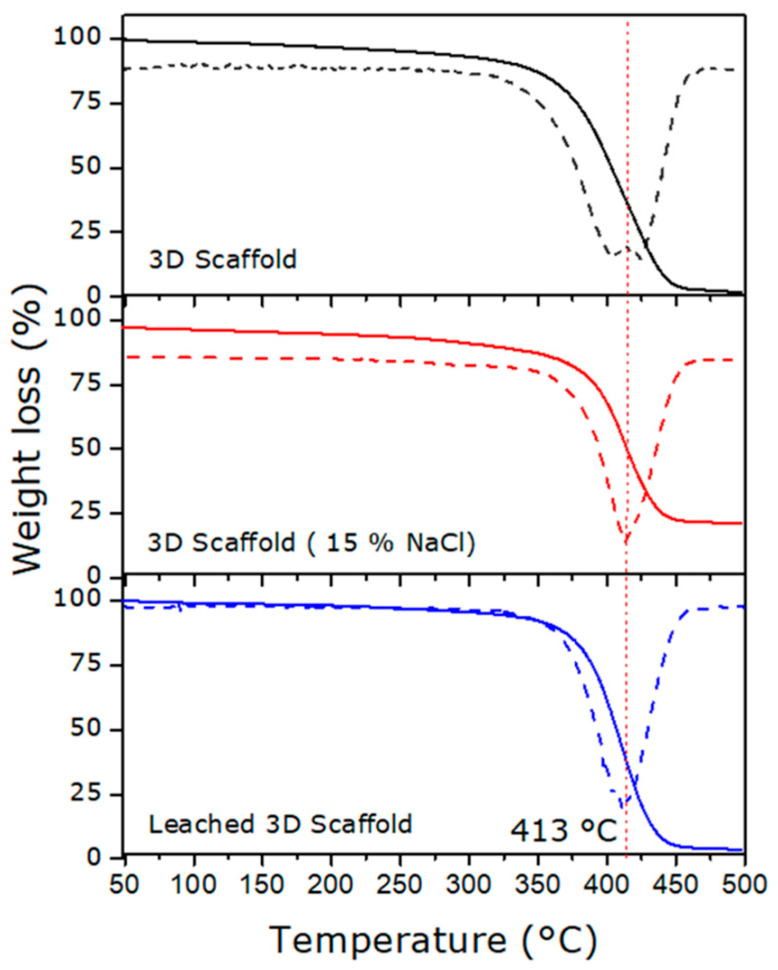
TGA thermograms of 3D scaffolds fabricated without porogen (no salt), with 15% NaCl, and after the leaching process. Solid line corresponds to TGA curves, and dashed to DTGA.

TGA was conducted on 3D scaffolds under three conditions: without salt particles, with 15% *w*/*w* salt particles, and after leaching. This technique enables the identification of potential differences in the thermal behavior of the materials, which may be associated with structural modifications and the presence of residual NaCl crystals within the samples. The thermograms of the three analyzed samples are shown in Figure 7.

All samples exhibited a thermal profile characterized by a moderate initial mass loss between 100 and 300 °C, primarily attributed to the early decomposition of DMAEMA side chains [43]. Subsequently, pronounced thermal degradation was observed starting at approximately 413 °C, corresponding to the breakdown of the polymer backbone, release of high-molecular-weight saturated and unsaturated aliphatic fragments, and carbonization, accompanied by CO and CO_2_ emission [44]. The presence of a single degradation step in all samples suggests the formation of a homogeneous copolymeric structure, characteristic of controlled radical polymerization routes such as PET-RAFT [45].

The sample containing salt exhibited a residual mass below 20%, attributable to the combined presence of residual carbon from pyrolysis and NaCl particles [46]. In contrast, the salt-free and leached samples showed residual masses below 5%, indicating almost complete polymer degradation, negligible salt retention, and residues primarily associated with carbon content. Overall, these results demonstrate the effectiveness of the leaching process in removing the inorganic porogen.

To complement the TGA results and further assess the thermal behavior of the polymeric network before and after porogen removal, a DSC analysis was performed on the salt-free and leached scaffolds. As shown in Figure S4, both samples exhibited a glass transition temperature (Tg) close to 8 °C (8.2 °C and 8.5 °C, respectively), indicating that the leaching process did not significantly alter the polymer structure. These glass transition temperature (Tg) values are consistent with those previously reported for DMAEMA/PEGDA-based formulations used to fabricate biocompatible hydrogels [43].

Since the Tg is well below physiological temperature (37 °C), the scaffolds remain in a viscoelastic state under these conditions, which may enhance their flexibility and mechanical adaptability under moderate loads and micromovements. This behavior is particularly advantageous for trabecular bone regeneration, where temporary mechanical support and the promotion of favorable cell–material interactions are essential [4].

### 3.4. Mechanical Characterization of Scaffolds

One of the main objectives of this study was to evaluate the mechanical performance of the scaffolds and compare it with that of human trabecular bone. Mechanical characterization was conducted via compression testing on three types of samples: solid cylinders, cylinders with channels, and cylinders with channels and pores. For these tests, six cylindrical specimens of each sample type were subjected to uniaxial compression until fracture. The loading rate (0.5 mm/min) exceeds the quasi-static strain rate limit of 1 s^−1^, indicating that the tests should be treated as dynamic analyses.

**Figure 8:**
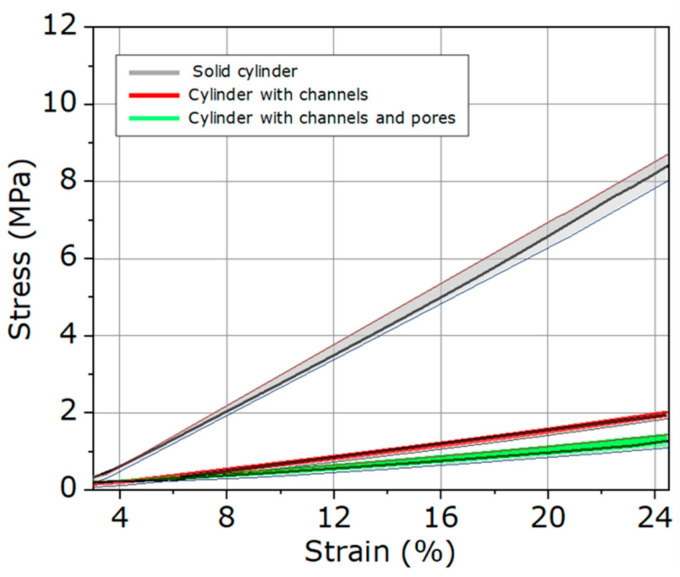
Stress–strain plot of the scaffolds with solid structures, cylinder with channels, and cylinder with channels and pores.

In all cases, a preload of 5 N and preconditioning protocols (20 cycles, until 5% strain) were applied to eliminate potential residual stresses. The results of the compression tests are summarized in Table 1 and illustrated in Figure 8. The graph depicts the shaded regions representing the maximum and minimum stress–strain ranges for each sample type, while the black lines show the average response of the six specimens tested.

Mechanical characterization revealed statistically significant differences (*p* < 0.05) in scaffold properties across architectures. The Young’s modulus of solid scaffolds (0.41 ± 0.03 MPa) was substantially higher than that of the scaffolds with channels (0.14 ± 0.02 MPa) and those incorporating both channels and pores (0.09 ± 0.01 MPa). This progressive reduction in stiffness was expected, as the introduction of channels and pores creates regions of lower density and increased compliance, which are advantageous for mimicking the porous structure of trabecular bone [4].

It is also noteworthy that the Young’s modulus values obtained for the scaffolds fall within the lower end of the stiffness range reported for trabecular bone (0.1–16 MPa) [47]. Regarding maximum fracture stress, the solid scaffolds reached 10.36 ± 0.49 MPa, decreasing to 2.61 ± 0.27 MPa for scaffolds with channels and 2.01 ± 0.33 MPa for scaffolds with channels and pores. Although a significant reduction in strength was observed, these values remain within the reported range of apparent compressive strength for trabecular bone (0.1–30 MPa) [4].

In terms of maximum strain, scaffolds with channels and pores exhibited the highest deformation (31.68 ± 1.44%), indicating greater flexibility and a greater capacity to absorb energy before fracture. This property is crucial in bone tissue engineering applications, as it enables the scaffold to adapt to micro-movements and local loads without undergoing premature failure [9].

#### 3.4.1. Enzymatic Degradation with Lipase

To evaluate the degradability of the porous 3D scaffolds under simulated enzymatic conditions, the materials were subjected to prolonged lipase treatment in PBS (pH 7.4) for 30 days. These conditions were designed to mimic an environment in which enzymes present under normal physiological conditions could accelerate the degradation of the polymeric matrix. To quantify this process, the samples were weighed (in triplicate) before and after treatment, following a 24 h vacuum-drying protocol. Based on these measurements, the weight loss during the analysis period can be calculated.

**Figure 9:**
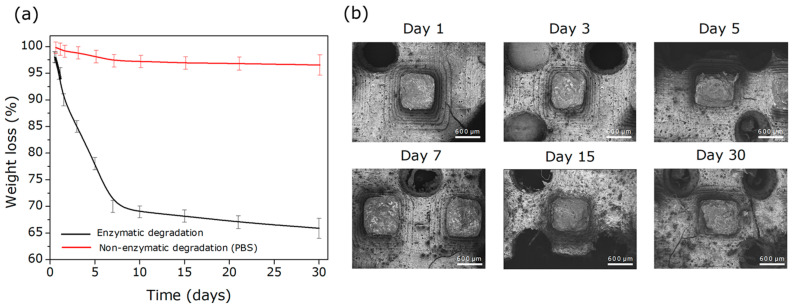
(**a**) Percentage of mass loss, and (**b**) FE-SEM micrographs of the 3D scaffolds during 30 days of incubation in enzymatic medium.

As shown in Figure 9a, the scaffolds exhibited progressive mass loss over 30 days, resulting in a total decrease of approximately 33% relative to the initial mass. An accelerated degradation phase was observed during the first week (~30%), likely due to the rapid diffusion of the medium into the most accessible domains of the material. As a control, scaffolds incubated only in PBS solution (pH 7.4, without enzymes) showed minimal mass loss of 3.4%, attributable to swelling-leaching of unstable fractions and limited non-enzymatic hydrolysis [48]. This marked difference confirms that the main degradation process is catalyzed by lipase rather than by the aqueous medium alone.

After the initial phase, the degradation rate decreased considerably, entering a slower and more sustained degradation stage that persisted until day 30, indicating a biphasic degradation profile rather than a purely controlled process. This behavior is characteristic of partially degradable polymeric materials, where factors such as the three-dimensional architecture, swelling capacity, crystallinity, hydrophobicity, polymer chain length, and water diffusivity limit the penetration of the enzymatic medium and the accessibility to hydrolysis-susceptible bonds. The observed degradation profile also indicates a homogeneous crosslinked polymeric network, in which, after the removal of more labile fractions, the remaining structure exhibits greater resistance to enzymatic attack.

Overall, these results demonstrate a biphasic biodegradation behavior, combining an initial burst phase followed by a sustained degradation stage, which is desirable for tissue engineering applications requiring gradual reduction in structural support in synchrony with tissue regeneration.

Complementing this quantitative analysis, morphological observations were performed using FE-SEM on the treated scaffolds. As shown in Figure 9b, the micrographs reveal evident surface modifications, including increased roughness, loss of definition at the porous edges, and the appearance of microfractures and irregular secondary pores. These alterations reflect the progressive effects of enzymatic hydrolysis, particularly in regions with high exposure or reduced polymer crosslinking density. Despite these surface modifications, the overall scaffold structure remained intact, with no evidence of structural collapse. This finding is particularly relevant for tissue engineering applications, where a degradation rate sufficiently slow to maintain mechanical support during the early stages of regeneration, yet active enough to allow cell integration and eventual biomaterial resorption, is highly desirable [49].

**Figure 10:**
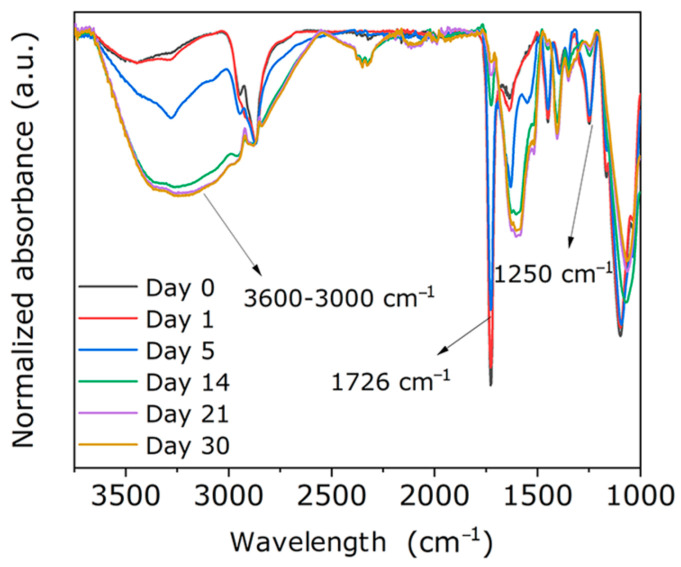
ATR-FTIR spectra of the porous 3D scaffold incubated in an enzymatic medium containing lipase for different periods (0–30 days).

Taken together, these results confirm that the scaffold exhibits progressive and structurally stable enzymatic biodegradability, reinforcing its potential as a biofunctional platform adaptable to physiological environments. To further elucidate the degradation mechanism, ATR-FTIR spectra of the samples incubated in the enzymatic medium for different time intervals were analyzed (Figure 10).

The ATR-FTIR spectra show progressive variations in the characteristic polymer bands, evidencing the hydrolytic action of lipase on the ester bonds of the matrix. Specifically, a gradual decrease was observed in the band associated with the C=O stretching of the ester groups (1726 cm^−1^), which can be attributed to the ester bond cleavage and the subsequent release of shorter-chain fragments or terminal carboxylic groups [50].

Additionally, the region between 3600 and 3000 cm^−1^ showed broadening and a relative increase in intensity, associated with the formation of hydroxyl (–OH) groups generated during hydrolysis, as well as with greater water absorption in the degraded structure [51]. Likewise, the decrease in the band around 1250 cm^−1^, associated with C–O–C vibrations of the polymer backbone, confirms bond cleavage within the macromolecular network [50]. Skrobot et al. [52] reported similar findings in polymer networks based on dimer acid macromonomers with methacrylic functionality, demonstrating that lipase incubation induces the cleavage of ester bonds as evidenced by ATR-FTIR analysis, particularly in bands around 1730 cm^−1^ and within the 1250–1020 cm^−1^ region, attributed to the formation of –OH and –COO^−^ groups after hydrolysis.

Overall, these spectral changes support the proposed controlled surface degradation mechanism, consistent with the gravimetric (% mass loss) and morphological observations. Lipase initially acts on the more accessible regions of the matrix, promoting ester bond cleavage and generating a progressively more hydrophilic and rougher surface. As degradation advances, hydrolysis proceeds at a slower rate, limited by diffusion of the enzymatic medium into the scaffold’s inner regions.

#### 3.4.2. Biological Characterization

This analysis is critical for determining whether the material is suitable for biomedical applications, particularly those in direct contact with living tissues. To this end, cytocompatibility assays were conducted following standardized protocols. These tests are designed to identify potential adverse effects resulting from direct material–cell contact or the release of soluble compounds from the material into the cellular environment. Assessing these parameters is essential for validating the material’s biocompatibility and its potential use in clinical applications or tissue engineering.

##### Evaluation of 3D Scaffold Cytocompatibility

**Figure 11:**
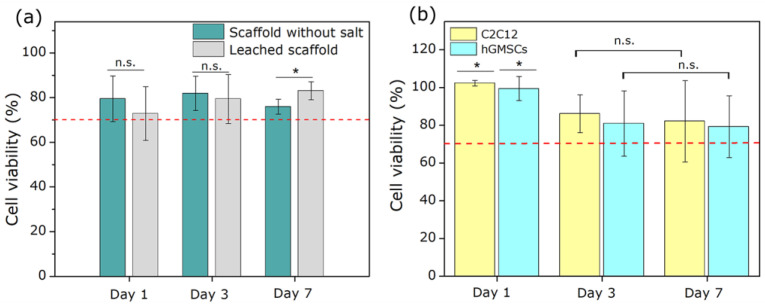
Cell viability of the (**a**) scaffold without salt and the leached scaffold with MC3T3-E1 cells, and (**b**) porous 3D scaffolds using C2C12 and hGMSCs cells at 1, 3, and 7 days of culture. The red dotted line represents the 70% viability threshold according to ISO 10993-5. (* *p* < 0.05; n.s.: not significant).

For the cell assays, the MC3T3-E1 cell line was selected because it provides a reliable model for predicting cellular behavior related to osteoconduction, including cell adhesion and cytocompatibility—key parameters for evaluating the material as a potential clinical biomaterial candidate [53]. The results of this analysis are presented in Figure 11a.

As shown in the graph, no statistically significant differences in cell viability were observed between the scaffold without salt and leached scaffolds on days 1 and 3. However, on day 7, a statistically significant increase in viability was observed for the porous scaffold. The recorded values remained above 70% cell viability at all times, thus meeting the requirements established by ISO 10993-5 for materials intended for biomedical applications. This trend suggests that removal of residual salt and generation of interconnected porosity not only preserved the cytocompatibility of the polymeric network but may also have enhanced cell viability and metabolic activity over time.

In addition to the osteoblastic MC3T3-E1 model, cytocompatibility assays were conducted using C2C12 murine myoblasts and human gingival mesenchymal stem cells (hGMSCs) to evaluate the scaffold’s compatibility in distinct cellular contexts with potential for osteogenic and myogenic differentiation. These cell lines provide a preliminary assessment of the biological versatility of the porous 3D scaffold by addressing not only its biocompatibility but also its capacity to support other clinically relevant cell types for regenerative applications [54,55].

As shown in Figure 11b, both C2C12 and hGMSCs exhibited cell viability values above 80% at 7 days, indicating good cellular tolerance to the material. In both cases, no statistically significant differences were observed between days 3 and 7, suggesting that the scaffold does not induce cytotoxic responses or progressive loss of cell viability over time. For hGMSCs, these findings are particularly relevant given the cells’ high sensitivity to microenvironmental conditions and substrate properties; their sustained viability further supports the scaffold’s potential as a supportive structure for complex regenerative applications [56].

These results are consistent with those obtained with MC3T3-E1 cells, demonstrating that, despite a slight drop in viability observed on day 7 for that specific line, the porous scaffold remains compatible with various cell types and meets the cytocompatibility standards established by ISO 10993-5. Collectively, this evidence supports the scaffold’s potential as a platform for future studies involving cell differentiation and the regeneration of osteogenic or musculoskeletal tissues.

##### Cytocompatibility of 3D Scaffold Extracts

**Figure 12:**
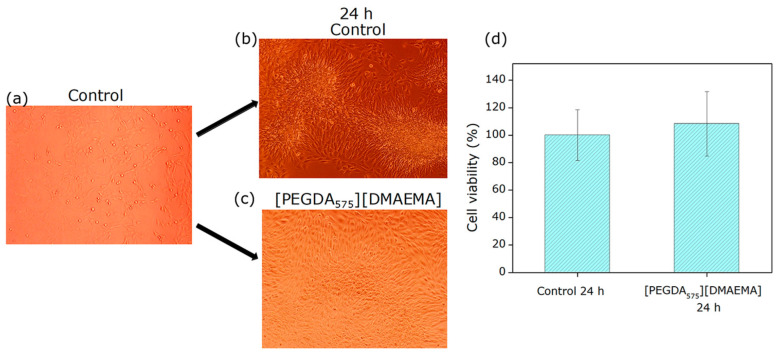
Optical micrographs of (**a**) control plate with C166-GFP cells, (**b**) control plate after 24 h of incubation, (**c**) plate with 3D scaffold extracts after 24 h of incubation, and (**d**) cell viability quantification using the Alamar Blue HS^®^ reagent kit (Thermo Fisher Scientific Inc., Waltham, MA, USA).

The leached samples were evaluated using the in vitro extract cytocompatibility assay described in ISO 10993-5 for the biological assessment of medical devices. In this procedure, each sample was placed in a culture plate containing the culture medium, alongside a control consisting of an empty well with the same medium, and incubated for 24 h. Subsequently, three aliquots of the extract were collected and mixed into a culture plate containing GFP-labeled C166 endothelial cells that had been growing for 24 h (Figure 12a).

As shown in the optical micrographs in Figure 12b,c, a high density of viable cells was observed in both the control wells and the wells containing extracts from the 3D scaffold. Notably, cell morphology appeared comparable to that of the control, with no detectable alterations. Metabolic activity was further confirmed by fluorescence quantification using the Alamar Blue HS^®^ reagent kit. The results of these analyses are presented in Figure 12d. The graph shows no statistically significant differences between the control and extracts from the DMAEMA:PEGDA_575_-based scaffold, indicating that the scaffold does not release cytotoxic compounds into the culture medium.

These findings are critically important for future research, as the development of materials intended for direct contact with human tissues requires rigorous safety evaluation, including both extract-based and direct contact cytocompatibility assays. Such tests are essential and mandatory to ensure safety before in vivo studies. Based on these positive outcomes, the incorporation of biodegradable PLGA nanoparticles loaded with insulin into the 3D scaffold is proposed to enhance its osteoinductive potential.

### 3.5. Preparation of 3D-Printed Scaffolds Loaded with PLGA–Insulin NPs

Insulin-loaded poly(lactic-co-glycolic acid) (PLGA) nanoparticles (NPs) were prepared using the double-emulsion solvent evaporation method (W1/O/W2) described by Santander et al. [29]. The PLGA employed had a molar ratio of 75/25 D, L-lactide/glycolide, and the internal aqueous phase of the loaded NPs was replaced with a 3% *w*/*w* solution of human insulin to achieve drug encapsulation. Subsequently, the porous scaffold was immersed in an aqueous suspension containing the loaded NPs, enabling their incorporation into the scaffold’s internal cavities. This process was monitored by FE-SEM to confirm nanoparticle distribution.

#### 3.5.1. Characterization of PLGA–Insulin NPs

**Figure 13:**
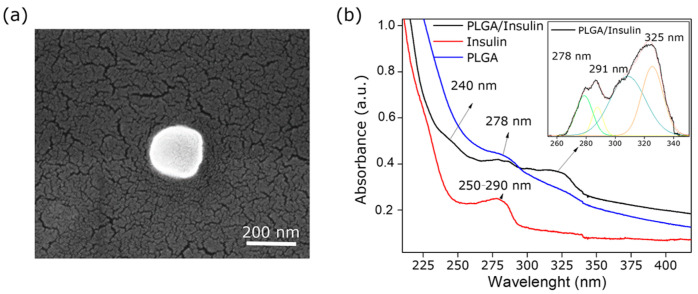
(**a**) FE-SEM micrograph of PLGA–Insulin NPs, and (**b**) absorbance spectra of PLGA/insulin NPs, PLGA NPs, and human insulin.

The size and polydispersity index (PDI) of the nanoparticles were determined by dynamic light scattering (DLS) experiments in milliQ water. As shown in Figure S5a, FE-SEM revealed an average particle diameter of 157.9 ± 21.6 nm, while Figure S5b presents the DLS results, which yielded an average size of 159.5 ± 4.6 nm and a PDI of 0.19, indicating a relatively narrow size distribution in the aqueous suspension [57]. Figure 13a further confirms the spherical morphology of the nanoparticles, consistent with previously reported sizes and shapes [29].

Finally, absorbance spectra were analyzed for three solutions: a 0.2% *w*/*v* PLGA/insulin NPs solution after 24 h of equilibration, a 0.2% *w*/*v* PLGA nanoparticle solution, and a 3% aqueous solution of human insulin, as shown by the blue line in Figure 13b. Two PLGA characteristic bands at 278 nm and 325 nm were also observed in the spectra [29]. In contrast, the red line displays a broad peak between 250 and 290 nm, typical of insulin spectra at high concentrations. The black line shows the absorbance of the PLGA–Insulin NPs, with previously identified PLGA signals evident alongside new signals attributable to insulin. In this case, an absorbance peak is observed at 291 nm, together with a maximum below 240 nm, corresponding to the chromophore protein groups of insulin [29]. This spectral shift was explained by Yanti et al. [58], who reported that at low concentrations, insulin exhibits variations in its characteristic absorbance peaks due to changes in the electronic environment of its chromophore groups, thereby altering the energies of molecular transitions.

#### 3.5.2. Incorporation of PLGA–Insulin NPs into the 3D Scaffold

**Figure 14:**
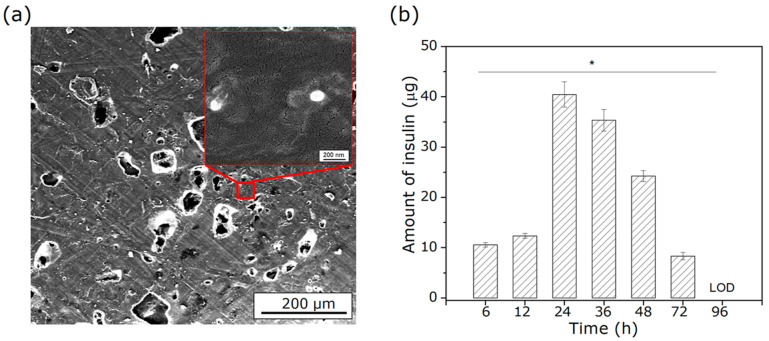
(**a**) FE-SEM micrographs of the porous scaffold with PLGA–Insulin NPs, and (**b**) quantification of insulin release determined by spectrophotometry. (* *p* < 0.05).

The successful incorporation of insulin-loaded PLGA nanoparticles (NPs) into the porous 3D-printed scaffolds was confirmed by FE-SEM analysis. As shown in Figure 14a, the presence of NPs on the surface of the porous 3D scaffold was confirmed, indicating successful incorporation of the NPs. To evaluate insulin release, UV-Vis absorbance measurements were performed in PBS (10 mM, pH 7.4) over 96 h. The results revealed a controlled and sustained release profile consistent with the polymeric system’s structural design. A marked increase in insulin concentration was observed during the first 24 h, attributed to an initial burst release phenomenon typical of PLGA-based systems and associated with insulin located on the surface or in the outer regions of the NPs [59]. This initial release is considered advantageous as it may create a favorable microenvironment for early stimulation of osteoinductive processes, thereby accelerating bone regeneration [60]. According to Figure 14b, the maximum insulin release occurred at 24 h (40.45 ± 0.45 µg), with sustained release maintained for up to 72 h.

This behavior indicates that the PLGA matrix continues to facilitate controlled drug diffusion throughout the degradation process. After 96 h, insulin concentrations fell below the limit of detection (LOD). Comparable studies on porous hydroxyapatite scaffolds loaded with PLGA–Insulin NPs, conducted by Wang et al. [61], reported that using the same insulin concentration in the NP formulation resulted in the release of approximately 62 µg of insulin within the first 24 h, followed by sustained release for at least six days. This release profile is particularly relevant for applications in bone tissue engineering, where continuous biochemical signaling is required during the early stages of trabecular bone regeneration and remodeling [62]. Due to its ability to stimulate osteoblastic proliferation and differentiation, insulin remains bioavailable at physiologically effective concentrations in the local environment during this critical period.

#### 3.5.3. Final Assessment of Cell Adhesion Under Dynamic Bioreactor Conditions

**Figure 15:**
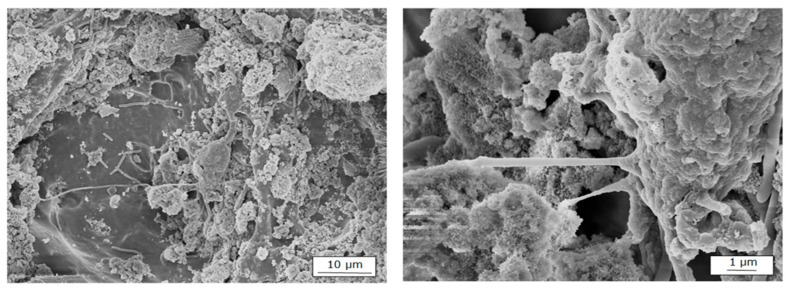
FE-SEM images showing cell adhesion on the material surface. (**Left**) Overview of cell morphology and spatial distribution. (**Right**) Higher-magnification image revealing lamellipodia and filopodia extending from the cell surface, indicating active interaction with the substrate.

To complement the cytocompatibility studies conducted under static conditions, a dynamic culture system was implemented using a custom-designed bioreactor that maintained the porous 3D scaffolds under controlled constant agitation environment. This dynamic environment promotes efficient nutrient and gas exchange, more closely mimicking the physiological microenvironment that the biomaterial would encounter in vivo. hGMSCs were used in this experiment due to their ability to adhere, proliferate, and differentiate into osteogenic tissues, making them a suitable cellular model for evaluating the biofunctionality of the scaffolds in bone regeneration applications [63]. Cell adhesion was initially assessed by FE-SEM to examine cell morphology and distribution on the scaffold surface (Figure 15). Subsequently, confocal microscopy analysis was performed using phalloidin staining for the cytoskeleton and Hoechst 33342 for nuclei, allowing confirmation of cytoskeletal organization and evaluation of cell spreading. Micrographs revealed that mesenchymal stem cells adhered to the surface of the 3D scaffold, exhibiting an extended morphology and forming cytoplasmic extensions, including filopodia and lamellipodia, which facilitate anchorage. These observations confirm effective cell–material interactions and suggest that the scaffold surface roughness and interconnected porosity provide a favorable microenvironment for cell proliferation.

**Figure 16:**
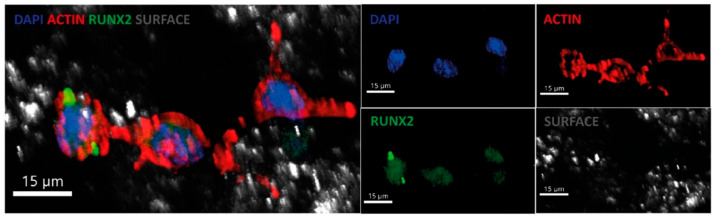
Immunofluorescence staining of MC3T3-E1 pre-osteoblastic cells cultured on the material surface. Nuclei were stained with DAPI (blue), F-actin with Rhodamine–Phalloidin (red), and the osteogenic marker RUNX2 with Alexa Fluor^®^ 488-conjugated antibody (green). The substrate surface is shown in gray. Representative confocal micrographs at 40× magnification are shown.

Confocal microscopy confirmed the viability and active adhesion of hGMSCs. The nuclei were stained in blue with Hoechst 33342, actin filaments were visualized in red using rhodamine–phalloidin (Cytoskeleton, Inc.), and expression of the transcription factor RUNX2 was detected in green using a recombinant monoclonal anti-RUNX2 antibody conjugated to Alexa Fluor^®^ 488 (Figure 16). At 10× magnification, cells adhered to the 3D scaffold are clearly visible. In contrast, at higher magnification (63×), nuclear colocalization of RUNX2 is distinctly observed.

It is worth noting that RUNX2 expression indicates early activation of the osteogenic pathway [64]. The organized arrangement of actin fibers suggests stable anchorage, while the elongated morphology and formation of interconnected cytoplasmic extensions indicate active cell–cell communication and matrix exploration [65]. These findings are directly associated with the scaffold roughness and interconnected porosity, which facilitate multipoint attachment, combined with the favorable osteogenic effect of the osteogenic agent (insulin), thereby promoting cell spreading and enhancing biofunctionality [66]. Overall, this behavior is further reinforced by the bioreactor’s dynamic conditions, which more accurately mimic the in vivo physiological microenvironment. This critical factor is particularly relevant in bone regeneration, where cells must adapt to irregular and porous surfaces.

## 4. Conclusions

This work effectively generated 3D-printed porous scaffolds via DLP using a DMAEMA/PEGDA_575_-based PET-RAFT resin, integrating NaCl as a porogenic agent and insulin-loaded PLGA nanoparticles as a bioactive component. This approach yielded structures with excellent print fidelity, a uniform trabecular architecture, and a highly interconnected porous network, including pore sizes of approximately 70 µm and a total porosity of approximately 57%, suitable for bone tissue engineering applications.

Physicochemical and thermal investigations confirmed successful polymerization via FTIR, Raman spectroscopy, and TGA. FE-SEM and micro-CT were used to analyze structural integrity and porosity after leaching and sterilization, while enzymatic and non-enzymatic degradability were assessed alongside swelling assays, demonstrating the adequate behavior of the 3D-printed scaffolds.

On the other hand, the scaffolds exhibited adjustable properties through architectural design, with elastic moduli and compressive strengths comparable to those of trabecular bone, thereby improving their mechanical compatibility with the host tissue.

Biological investigations demonstrated notable cytocompatibility, with cell viability exceeding 80% across multiple pertinent cell lines (MC3T3-E1, C2C12, hGMSCs, and C166-GFP), in compliance with international standards (ISO 10993-5). The homogeneous integration of PLGA–Insulin nanoparticles enabled continuous release of the osteoinductive factor, characterized by an initial uptake followed by sustained release over 72 h, establishing a favorable bioactive environment for the early stages of bone regeneration. Furthermore, dynamic cultures in a bioreactor showed improved cell adhesion, cytoskeletal organization, and RUNX2 expression, confirming the early activation of osteogenic pathways.

This study serves as an initial demonstration of a PET-RAFT-based DLP-printed scaffold that simultaneously offers structural support and controlled spatiotemporal insulin release through embedded PLGA nanoparticles, thereby establishing a dual-function strategy that integrates fabrication precision with biochemical stimulation. These results establish this strategy as a flexible and therapeutically relevant method for next-generation bone tissue-engineering scaffolds and targeted therapeutic delivery.

## Figures and Tables

**Table 1:** Young’s modulus, maximum fracture stress, and strain of scaffolds with solid structure, a cylinder with channels, and a cylinder with channels and pores.

	Young’s Modulus (MPa)	Maximum Fracture Stress (MPa)	Maximum Strain (%)
Solid cylinder	0.41 ± 0.03	10.36 ± 0.49	27.97 ± 5.60
Cylinder with channels	0.14 ± 0.02	2.61 ± 0.27	24.60 ± 2.23
Cylinder with channels and pores	0.09 ± 0.01	2.01 ± 0.33	31.68 ± 1.44

## Data Availability

The data supporting this study’s findings are available from the corresponding author upon reasonable request.

## Supplementary Materials

The following supporting information can be downloaded at: https://www.mdpi.com/article/10.3390/polym18101184/s1, Figure S1: Resin deposited on the build plate; Figure S2: Pore size histogram determined by FE-SEM; Figure S3: Histogram of pore size obtained from 3D micro-CT analysis; Figure S4: DSC thermograms of the 3D scaffold before and after the leaching process; Figure S5: Particle size distribution of insulin-loaded PLGA nanoparticles determined by FE-SEM and DLS; Table S1: Rheological parameters of the commercial resin and the [PEGDA_575_][DMAEMA]-based resin, including Bond number calculations.
