# Supplementary material for: Engineering Porous PET-RAFT Scaffolds with PLGA–Insulin Nanoparticles: Advancing Bone Tissue Regeneration Through Additive Manufacturing
Source: Polymers (Basel). 2026 May 12;18(10):1184. doi: 10.3390/polym18101184 (PMC13211159; doi:10.3390/polym18101184)
Supplement: Supplementary file 1 [file polymers-18-01184-s001.zip › polymers-4280269-supplementary.pdf]

Supplementary material

S1. Synthesis and Printability Evaluation of the Resin

Both the monomer (DMAEMA) and the crosslinker ($PEGDA_{575}$) were purified prior to resin formulation by passing them through a basic alumina column (aluminum oxide 90 active basic, 0.063–0.200 mm) to remove polymerization inhibitors, such as MEHQ, thereby facilitating effective curing. The resin formulation was prepared using a molar ratio of [100]:[400]:[1]:[0.01]:[20]:[0.01] corresponding to [DMAEMA]:[$PEGDA_{575}$]:[BTPA]:[Eosin Y]:[triethanolamine (TeOH)]:[Orange G (OG)], respectively. These ratios were selected based on previously optimized PET-RAFT systems developed by our research group, ensuring a balance between controlled polymerization kinetics, light absorption, and printability under DLP conditions.

The reaction mixture was prepared in amber glass vials, sealed, and kept in the dark. The mixture was vortexed for 5 min, then sonicated for 20 min to ensure homogeneity.

In parallel, sodium chloride (NaCl) particles were prepared as a porogen agent. NaCl crystals were first ground using a blender and subsequently refined using an agate mortar. The resulting particles were sieved to obtain a size range of 63–75 µm (mesh No. 300 and No. 200, respectively), ensuring adequate suspension within the resin. After sieving, the NaCl particles were incorporated into the resin at 15 % w/w and sonicated for 30 min prior to printing.

Two primary analyses were conducted in this study to evaluate the printability of the resin: the determination of the dimensionless Bond number (B_o, **Eq. S1**), and contact angle measurements between the resin and the metallic build plate. The viscosity of the bare resin was measured using a rheometer via dynamic oscillatory shear tests. Surface tension measurements of the bare resin were also performed, and its density was determined using the gravimetric-volumetric method. These parameters were used to calculate the Bond number according to the following equation:

$$\mathrm{B_o} = \Delta\rho g L^2/\sigma \qquad \textbf{Eq. (S1)}$$

Where Δρ is the density difference between the liquid (resin) and the surrounding gas (air). In this case, the air density is negligible, so Δρ≈ρ of the fluid; g is the Earth's gravitational acceleration; L is the characteristic length of the system (in this case, the layer thickness, 25 µm); and σ is the surface tension whose values were obtained from the measurements reported in **Table S1** of the Supplementary Information. For contact angle measurements, 4 µL of liquid resin was placed onto the metallic printing platform and imaged using an optical tensiometer (Theta Optical Tensiometer). These studies were conducted in parallel with a commercial biocompatible resin, Raydent Crown & Bridge (Zortrax), used as a control.

Table S1. Rheological parameters of the commercial resin and the [$PEGDA_{575}$][DMAEMA] based resin. The Bond number was calculated using **Eq. 1s**.

Resin	Dynamic viscosity (mPa·s)	Surface tension (mN/m)	Density (g/mL)	Bond number (B_o)
Commercial	530.46 ± 0.013	30.75 ± 1.70	1.01 ± 0.008	1.01×10^{-4}
[$PEGDA_{575}$][DMAEMA]	18.70 ± 0.004	47.20 ± 0.54	1.13 ± 0.015	1.13×10^{-4}

S2. Scaffold design, DLP printing parameters, and post-processing

Scaffold models were designed using AutoCAD 2022 (Autodesk, Inc.). Cylindrical geometries incorporating vertically and horizontally aligned channels (channel diameter Ø = 1 mm) were generated. The scaffolds were fabricated using a DLP printing system with resin formulations containing either NaCl particles or none.

The printing parameters were as follows: a layer thickness of 25 μm and an exposure time of 120 s per layer. To enhance adhesion between the printed layers and the build platform, the first five layers were exposed for 200 s. Each model was printed five times to ensure reproducibility.

After printing, the cylindrical scaffolds were washed in isopropanol for 10 min, air-dried, and subjected to an additional UV curing step (λ = 385–405 nm) for 30 min using a Mercury Plus 2-in-1 unit (Elegoo Inc.).

For scaffolds containing NaCl particles, an additional leaching process was performed to remove the porogen, as described in the Supplementary Material.

S3. Characterization of the Scaffold

S3.1. Incorporation of Porogen Agent and Leaching Process

Once the printing parameters were standardized, NaCl particles with sizes ranging from 63 to 75 μm were incorporated into the resin at 15 wt.% (8.5 vol.%), based on the respective densities of the resin and the salt. Printing was carried out using the same parameters as previously described, with the process paused every 20 minutes to prevent particle sedimentation. The printed cylinders were weighed, then immersed in a deionized water bath at room temperature for leaching. Samples were weighed at different time points (1, 3, 7, 12, 24, 36, and 48 hours of leaching) to construct a mass loss curve. After each time, the cylinders were removed from the bath, gently dried with absorbent paper to remove surface water, and degassed in a vacuum chamber (10^{-3} torr) for 24 hours. The optimal leaching time was determined from the mass-loss curves.

S3.2. Thermal Analysis

To evaluate the effect of the leaching process on the thermal stability of the porous 3D scaffolds, thermal analyses were performed. All experiments were carried out under a N_2 atmosphere with a constant flow rate of 50 mL/min to prevent oxidation during thermal decomposition. Three types of samples were analyzed: the scaffold without salt particles (control), the scaffold containing 15 wt.% NaCl, and the scaffold after leaching. For each sample, 5–10 mg was weighed and placed in platinum crucibles. The thermal protocol consisted of a heating cycle from 25 °C to 800 °C at 10 °C/min. Mass-loss (%) curves were recorded to identify decomposition events and inorganic residues after calcination. DSC analysis was performed on both the bare scaffold and the scaffold after leaching. The first heating cycle was recorded from −40 °C to 180 °C, followed by cooling, and a second heating cycle under the same conditions. The glass transition temperature was determined as the midpoint of the baseline shift in the thermogram.

S3.3. Uniaxial Compression Testing

Three types of solid cylindrical specimens were analyzed in this test: solid, with channels, and with channels and micropores (produced by salt leaching). Deformation was measured in a quasi-static compression test using flat plates that applied a

continuous, uniform load at 0.5 mm/min until either fracture or a maximum load of 2500 N was reached. Before mechanical testing, a preload of 5 N was applied to ensure optimal conditions, including proper alignment between the sample and the compression plates, specimen stabilization, and protection of the machine's sensors from sudden localized loads at the beginning of the test. In addition, pre-conditioning cycles were performed to release residual stresses induced during the manufacturing process. These consisted of 20 cycles with 5 % strain, and a cycling speed of 5 mm/min.

S3.4. Morphological Characterization of the Scaffold

FE-SEM analyzed surface morphology, while elemental composition was assessed using EDX. Furthermore, micro-CT analysis was performed on the porous 3D scaffold to analyze the intertal structure. The X-ray source voltage and current were set to 40 kV and 250 μA, respectively. Tomographic images were acquired at two different resolutions: 25 μm per voxel for a quick scout and 3 μm per voxel for a high-resolution scan. The 3D images were reconstructed using NRecon software. During reconstruction, standard correction and filtering parameters were applied to optimize image quality and enable reliable quantitative analysis. This included ring artifact correction and the application of a Hamming filter with a cut-off frequency set to 100% of the Nyquist frequency. Using these reconstructed images, representative volume elements (RVEs) were selected from different sectors of the cylinder to calculate local porosity (**Eq. S2**), determine pore volume, and estimate the Euler number of the internal scaffold architecture.

$$Porosity\ (\%) = \frac{Pore\ volume}{Pore\ volume\ + Matrix\ volume} * 100 \qquad \textbf{Eq. (S2)}$$

S4. Statistical analysis

Statistical analysis of the biological assays and physicochemical characterizations was conducted using OriginPro 2021. A one-way ANOVA test was applied with a significance level of $p < 0.05$. Before the analysis, assumptions of normality and homogeneity of variances were assessed using the Shapiro–Wilk and Levene's tests, respectively. When statistically significant differences were identified, post hoc comparisons were conducted using Tukey's HSD (Honest Significant Difference) test to determine which groups differed significantly. Data are presented in the graphs as mean ± standard deviation.

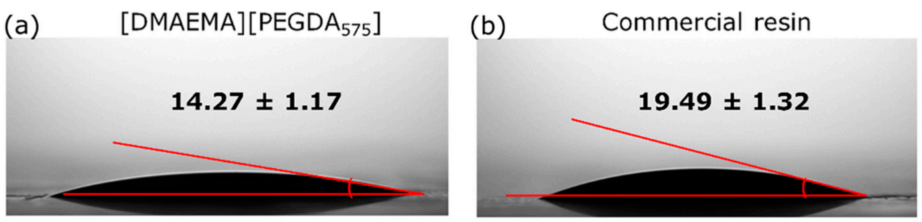

Figure S1. (a) [PEGDA$_{575}$][DMAEMA] based resin, and (b) commercial Raydent-Anycubic resin deposited on the DLP printer build plate.

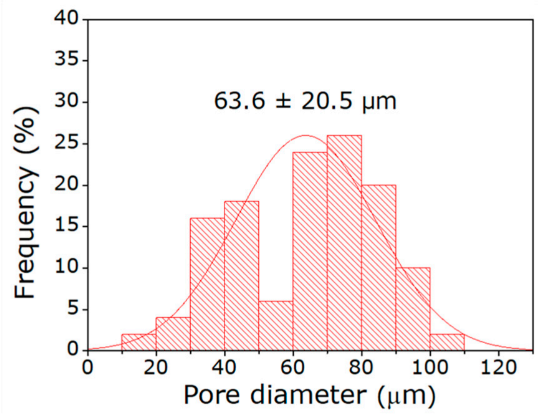

Figure S2. Pore size histogram determined by FE-SEM.

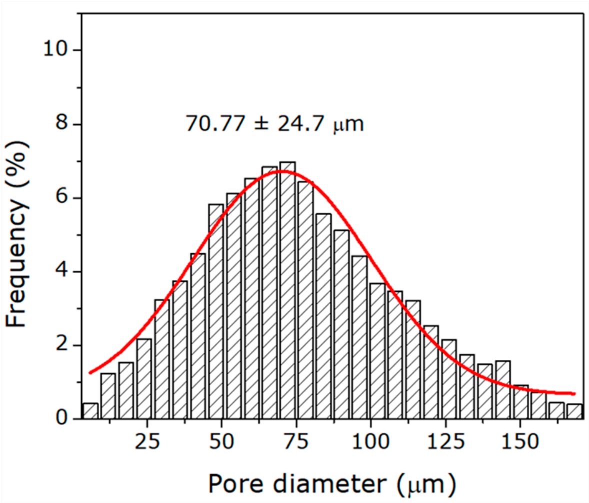

Figure S3. Histogram of pore size obtained from 3D micro-CT analysis.

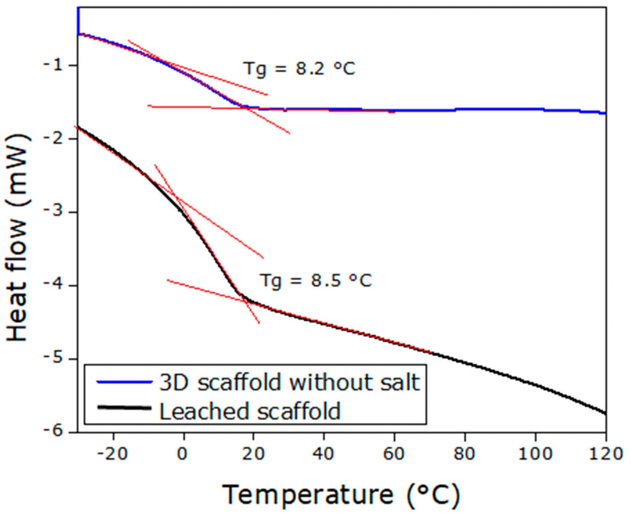

Figure S4. DSC of the 3D scaffold before and after the leaching process.

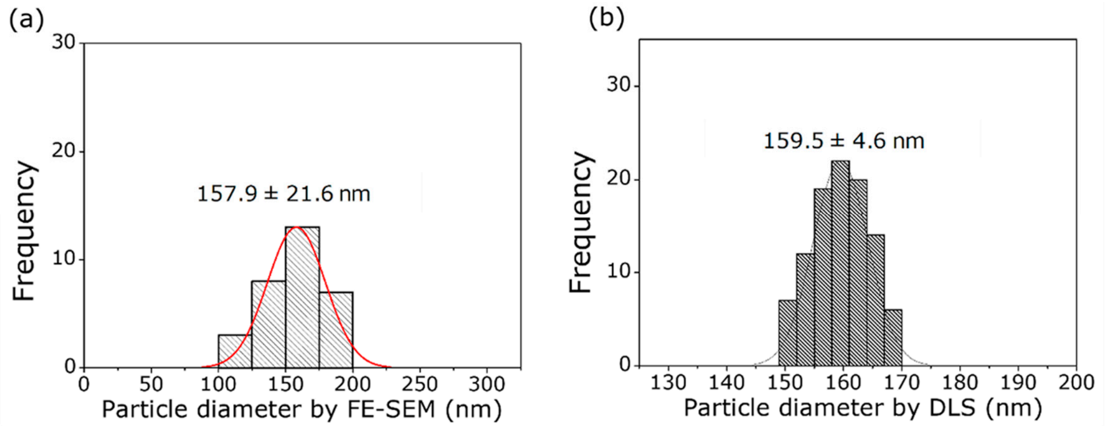

Figure S5. Particle size distribution of insulin-loaded PLGA nanoparticles determined by (a) FE-SEM and (b) DLS.
